# High Local and Systemic Expression of Pentraxin-3 in Anaplastic Thyroid Cancer

**DOI:** 10.3390/ijms262311335

**Published:** 2025-11-24

**Authors:** Andreea Bojoga, Pepijn van Houten, Martin Jaeger, Katrin Rabold, Birgitte Walgreen, Liesbeth van Emst, Dumitru Ioachim, Ilse van Engen-van Grunsven, Corin Badiu, Romana T. Netea-Maier

**Affiliations:** 1 Faculty of Medicine, Carol Davila University of Medicine and Pharmacy, 020021 Bucharest, Romania; andreea-cristiana.coriiu@drd.umfcd.ro (A.B.); corin.badiu@umfcd.ro (C.B.); 2 Department of Internal Medicine, Division of Endocrinology, Radboud University Medical Center, 6525 GA Nijmegen, The Netherlands; pepijn.vanhouten@radboudumc.nl (P.v.H.); martin.jaeger@radboudumc.nl (M.J.);; 3 Rheumatology Research Laboratory, Department of Internal Medicine, Radboud University Medical Center, 6525 GA Nijmegen, The Netherlands; 4 Department of Pathology, National Institute of Endocrinology C. I. Parhon, 011863 Bucharest, Romania; 5 Department of Pathology, Radboud University Medical Center, 6525 GA Nijmegen, The Netherlands; 6 Department of Endocrinology IV, National Institute of Endocrinology C. I. Parhon, 011863 Bucharest, Romania; 7 Faculty of Medicine, Iuliu Hatieganu University of Medicine and Pharmacy, 400129 Cluj-Napoca, Romania

**Keywords:** non-medullary thyroid cancer, anaplastic thyroid cancer, tumor-associated inflammation, pentraxin 3

## Abstract

Chronic inflammation plays a key role in cancer pathogenesis. Aggressive thyroid cancer is associated with immune infiltration and systemic inflammation. Long pentraxin 3 (PTX3) is an inflammatory protein implicated in tumor progression. This study evaluates PTX3 plasma levels in patients with non-medullary thyroid cancer (TC) compared to benign thyroid disease and investigates its tissue expression. We prospectively included 55 TC patients: 42 papillary, 3 follicular, 4 oncocytic, 4 anaplastic (ATC), and 2 poorly differentiated (PDTC). The control group consisted of 32 patients with benign thyroid disease. PTX3 plasma concentrations were measured by ELISA, and tissue expression of PTX3 and CD68 was analyzed using immunohistochemistry. PTX3 plasma levels did not significantly differ between TC and controls, but patients with PDTC and ATC had markedly higher concentrations. Tissue analysis showed strong PTX3 expression in three of four ATC cases in tumor and stromal cells, whereas benign and differentiated thyroid tissues exhibited minimal staining. CD68 expression was positive in ATC, indicating tumor-associated macrophage infiltration, but a few cells were double-positive for PTX3 and CD68. Our findings suggest a possible association between PTX3 and aggressive TC, particularly ATC. Further studies are needed to validate these findings and elucidate the cellular origin and functional role of PTX3.

## 1. Introduction

Patients with advanced metastatic radioiodine refractory non-medullary thyroid carcinoma (TC), particularly those with poorly differentiated (PDTC) and anaplastic thyroid cancer (ATC), have a poor prognosis [1]. ATC is one of the most aggressive human cancers, with rapid disease progression, a median survival of six months from diagnosis, and very limited treatment options [2]. Understanding the pathogenesis of aggressive TC is essential for identifying novel treatment targets.

Tumor-related inflammation is fundamental in cancer pathogenesis, with numerous studies showing the role of inflammation in the initiation, growth, and development of tumors in various cancer models [3]. This is also the case for TC. Abundant tumor-associated macrophage (TAM) infiltration in aggressive TC correlates with the presence of lymph node metastases, invasive disease, and poor prognosis [4], and aggressive forms of TC showed higher inflammatory parameters, such as interleukin-1 receptor antagonist and C-reactive protein, compared to healthy volunteers [5,6]. Additionally, an association of genetic variants linked to increased proinflammatory markers, such as interleukin-1β, with a higher susceptibility to develop TC and a reduced response to radioiodine treatment was reported [7]. We have shown that the proinflammatory cellular program of TAMs interacts with TC cells and that tumor cell-derived metabolites induce epigenetic and metabolic changes responsible for these interactions. Moreover, we have shown that the transcriptional and functional phenotype of TAMs is programmed even before these myeloid cells infiltrate the tumor and are present even at the level of the bone marrow progenitors [8].

Innate immune responses are triggered when damage-associated molecular patterns (DAMPs) are recognized by pattern recognition molecules (PRMs) [9]. One notable group of soluble PRMs is represented by pentraxins, evolutionarily conserved molecules with roles in innate immunity and inflammation, including the regulation of complement activation and pathogen opsonization [10].

Pentraxin 3 (PTX3), also known as Tumor Necrosis Factor-Stimulated Gene 14 protein (TSG-14), is a pleiotropic member of the long-pentraxin subfamily. It is produced locally at inflammation sites by various cell types, including myeloid, vascular, and lymphatic endothelial cells, and mesenchymal and epithelial cells, in response to diverse inflammatory signals [11]. The human *PTX3* gene expression is induced by proinflammatory cytokines such as IL-1β, IL-6, tumor necrosis factor (TNF), and toll-like receptors (TLR) agonists [9,11].

Recent in vivo and in vitro studies indicate that PTX3 is involved in cancer-related inflammation and plays a role in various aspects of cancer progression, including tumor onset, angiogenesis, metastatic spread, and cancer immune modulation [11,12,13,14]. Both tumor-promoting and tumor-suppressing activities have been reported for PTX3, with its function possibly varying depending on tumor type, cellular source, and surrounding environment [11]. The expression of the *PTX3* gene has been reported to be upregulated in different solid tumors, including ATC [11,15]. Higher circulating PTX3 concentrations have been reported in patients with malignant tumors, particularly in more advanced disease stages [13,16,17]. Intratumoral expression of PTX3 was increased in lung cancer, primary brain tumors, and hepatocellular carcinoma versus non-neoplastic tissue, and this was also associated with worse prognosis [17,18,19]. Overall, PTX3 appears to promote tumor progression in various models. However, the exact mechanism is not fully understood.

This study aims to evaluate the circulating concentrations of PTX3 in patients with TC compared to patients with benign thyroid diseases and to investigate whether these correlate with the histological TC forms, clinical parameters, and PTX3 expression in representative tissue samples from these patients.

## 2. Results

### 2.1. Clinical and Demographic Characteristics

In total, 87 patients were included: 32 in the control group (20 benign goiter and 12 chronic autoimmune thyroiditis) and 55 in the TC group (41 preoperatively, 14 with recurrent active disease) (Table 1). There was no significant difference in sex distribution (60.0% vs. 81.2% female, *p* = 0.650), age at the time of blood collection (55 years (48–79) vs. 62 years (49–81), *p* = 0.173), and BMI (27.5 ± 4.6 vs. 30.4 ± 6.7, *p* = 0.252) in the TC vs. control group.

### 2.2. PTX3 Plasma Concentrations

**Figure 1:**
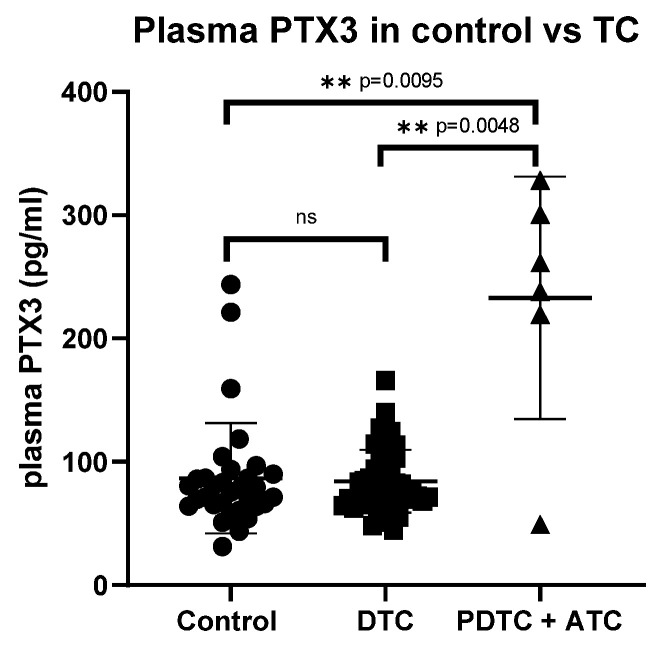
Comparison of PTX3 plasma concentrations in the control group and thyroid cancer. PTX3: pentraxin 3, TC: thyroid cancer, DTC: differentiated thyroid cancer, PDTC: poorly differentiated thyroid cancer, ATC: anaplastic thyroid cancer, ns: not statistically significant, ** statistically significant.

The PTX3 plasma concentrations in patients with ATC (269.4 pg/mL (224.2, 321.4)) and PDTC (155.5 pg/mL (49.3, 261.7)) were significantly higher than those of patients with differentiated TC (76.6 pg/mL (69.3, 103.2)) and control patients (73.5 pg/mL (64.4, 89.1)) (*p* = 0.004 and *p* = 0.009, respectively) (Figure 1). When analyzing PTX3 levels according to AJCC stage, the difference became statistically significant (*p* = 0.040) when poorly differentiated and anaplastic thyroid carcinomas (PDTC and ATC) were included. This significance was mainly driven by the higher PTX3 concentrations observed in PDTC and ATC cases, as the difference lost statistical significance after their exclusion.

Overall, there was no significant difference in PTX3 concentrations between patients with TC and controls (*p* = 0.275). Histological subtypes within differentiated tumors, disease extent, and sensitivity to RAI treatment had no significant effect on PTX3 concentrations, either when compared with the control group or when analyzed by subgroups (Table 1). No significant difference in plasma PTX3 levels was observed between the control and TC group after excluding patients with (background) lymphocytic thyroiditis from both cohorts (*p* = 0.671). Similarly, plasma PTX3 levels did not differ significantly between patients with recurrent active disease (after primary surgery), patients with TC before surgery, and controls. Age did not correlate significantly with plasma PTX3 levels in either the control group (r = 0.254, *p* = 0.061) or the TC group (r = 0.216, *p* = 0.233).

### 2.3. PTX3 Expression and Distribution in TC Tissue

**Figure 2:**
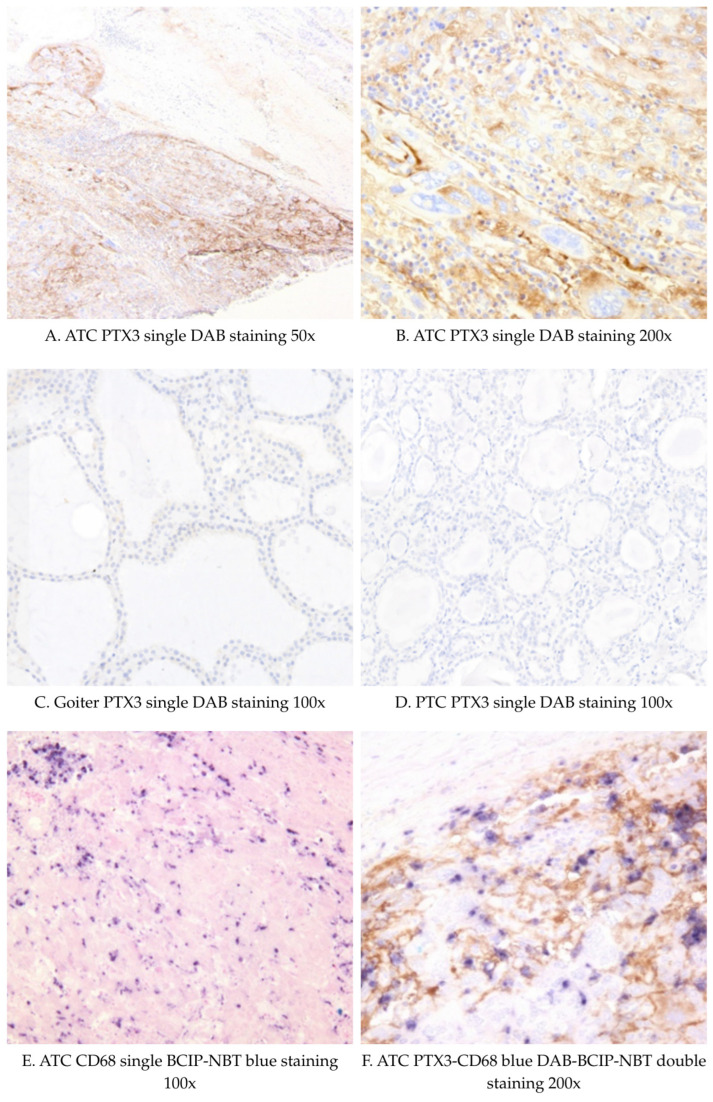
Representative IHC images of PTX3 (DAB staining) and CD68 staining (blue DAB-BCIP-NBT) in thyroid cancer and goiter tissues. Abbreviations: PTC: Papillary thyroid cancer; ATC: Anaplastic thyroid cancer; DAB: 3,3′-diaminobenzidine (DAB); BCIP/NBT: 5-bromo-4-chloro-3-indolyl phosphate/nitro blue tetrazolium.

We have further assessed PTX3 and CD68 expression by immunohistochemistry in tissue samples from 2 patients with goiter, 1 patient with papillary TC (PTC), and 4 patients with ATC (Figure 2). A summary of immunohistochemistry findings is presented in Supplementary Table S2. PTX3 staining was strongly positive in 3 out of 4 ATCs, predominantly intracellular with some interstitial staining. There was a higher number of infiltrating CD68-positive cells in ATC compared with the PTC and goiter samples. Nonetheless, there were only a few scattered CD68-positive cells, which also stained positively for PTX3, indicating a low co-localization. The PTX3-negative ATC patient (ATC4) had high plasma PTX3 levels but weak tissue PTX3 and CD68 staining. PTX3 staining was virtually absent in the PTC and goiter tissues, with sparse and faint staining. Given the limited number of ATC and PDTC samples, the immunohistochemical findings should be interpreted descriptively.

## 3. Discussion

This study provides novel insights into the expression and potential role of PTX3 in ATC. While overall no significant difference in plasma PTX3 concentrations was observed between patients with TC and benign thyroid disease, increased PTX3 expression was found in patients with ATC. This finding suggests a potential association between elevated PTX3 and the aggressive clinical phenotype of this cancer subtype. To our knowledge, this is the first study to evaluate PTX3 either in plasma or tissue in ATC.

These observations have several potential implications. Prior studies have suggested that PTX3 may reflect a more aggressive tumor phenotype in other cancers, indicating that it could also have prognostic relevance. A genome-wide analysis of ATC and other differentiated subtypes of TC indicated that *PTX3*, *COLEC12*, and *PDGFRA* were overexpressed in ATC while being underexpressed in follicular or PTC [15]. Another bioinformatic analysis of eight gene-expression profiles found a four-gene prognostic signature for PTC, which included *PTX3*, *PAPSS2*, *PCOLCE2*, and *TGFBR3*, which showed better performance in overall survival prediction than the American Joint Committee on Cancer (AJCC) staging system [20]. PTX3 tissue expression in PTC may be highly localized within the tumor microenvironment, particularly in specific cell populations or stromal areas. The absence of PTX3 staining in our PTC samples could be related to heterogeneity among PTC subtypes, differences in antibody specificity or sensitivity, or the small sample size analyzed. PTX3 is a component of the innate immune response that is produced at inflammatory sites, and elevated tissue PTX3 expression in tumor tissue does not necessarily translate into increased plasma concentrations, as circulating levels depend on both local secretion dynamics and systemic clearance. Concerning plasma PTX3 concentrations, Chiari et al. found no differences between 53 patients with benign or malignant thyroid nodules, but higher PTX3 concentrations in these patients than in healthy volunteers. The authors hypothesized that PTX3 overexpression could be associated with active phases of nodular remodelling [21]. In line with these results, Destek et al. found no significant difference in PTX3 plasma levels between patients with benign thyroid nodules (41 patients) and those with cytologically confirmed PTC (14 patients), regardless of nodule size or number [22]. Our study aimed to extend these investigations to patients with a broader spectrum of TC histological phenotypes, including patients with more advanced disease. Overall, our results yielded no significant difference in PTX3 plasma concentrations between the TC group and the group of patients with benign thyroid pathology, nor within the TC group, according to differentiated histological subtypes, presence and extent of active structural disease, or other aggressive features such as RAIR. However, the fact that ATC clearly showed increased concentrations of PTX3 in circulation and higher expression of PTX3 in the tissue samples aligns with previous research indicating that the role of PTX3 in cancer may be context-dependent, influenced by the specific cancer type [9].

The present findings raise important questions about the potential contribution of PTX3 in the pathogenesis of TC, particularly aggressive forms such as ATC. PTX3 has been reported to promote cell migration and invasion in various tumor models. The in vitro study by Kondo et al. demonstrated that high PTX3 levels produced by pancreatic carcinoma cell lines are linked to tumor progression and poor patient outcomes [23]. PTX3 enhances EGF-induced migration, invasion, and metastasis in head and neck squamous cell carcinoma cells [12]. Song et al. reported a significantly higher PTX3 immunostaining in hepatocellular carcinoma compared to normal adjacent liver tissue and showed that PTX3 promotes tumor invasion, cell proliferation, and epithelial–mesenchymal transition by in vivo xenograft experiments [17]. The high PTX3 expression we found in ATC tissues supports the hypothesis that PTX3 is involved in the inflammatory microenvironment of aggressive tumors, potentially contributing to macrophage infiltration and function [17]. In the ATC patients, there was an increased infiltration with CD68-positive macrophages, concordant with the high infiltration with TAMs previously reported [4], as well as a strong PTX3 expression in both tumoral cells and interstitial ATC tissues. However, immunohistochemically, PTX3 and CD68 staining were virtually absent in patients with goiter, and in those with PTC. These findings are in line with the high PTX3 expression at the tissue level in other tumoral tissues, such as gliomas, particularly high-grade anaplastic gliomas, and glioblastoma, unlike the low-grade tumors [19]. Interestingly, The Human Protein Atlas has published expression data of PTX3 in cell lines including TC, and reported higher amounts of PTX3 produced by the ATC-derived cell line 8505C, compared to ATC-derived cell line CAL-62, squamous cell TC derived cell line SW579, PTC derived cell lines (BCPAP, BHT-101, TPC-1), follicular TC derived cell lines (FTC-133, FTC-238) [24]. In the high-grade glioma tissue, PTX3 was expressed by both tumor cells and activated macrophages [19]. In our study, however, there was a low co-localization of the CD68 and PTX3 in the tumor microenvironment, suggesting that PTX3 was mainly produced by tumoral cells, other immune cells, or stromal cells rather than the CD68-positive macrophages that were highly infiltrating the tumors. Nonetheless, because only a single macrophage marker was examined for the immunohistochemistry, we cannot exclude with certainty the macrophage origin of PTX3. It is also possible that the intracellular and interstitial staining observed could represent cellular uptake or deposition in the extracellular matrix rather than actual production. Moreover, the finding of a high PTX3 plasma concentration in one patient in which the tissue expression of PTX3 was virtually absent suggests that PTX3 could also be even synthesized outside the tumor site, such as the liver, potentially upon the stimulatory effects of other systemic proinflammatory cytokines, such as IL-6, also reported to be increased in ATC [25].

Although additional markers such as CD163 (M2 macrophages), CD3/CD8 (T lymphocytes), or α-SMA (stromal fibroblasts) could provide a more comprehensive characterization of the tumor microenvironment, the present study was designed as an exploratory analysis with a limited sample size. Future studies incorporating these additional immune and stromal markers are warranted to further elucidate the cellular sources of PTX3 and its role within the thyroid cancer microenvironment.

Our study has some limitations. PTX3 plasma concentrations could reflect other systemic inflammatory conditions. Patients with ATC were significantly older than patients with benign thyroid disease, thus possibly harbouring endothelial dysfunction, atherosclerosis, with associated smoldering inflammation, hence potentially confounding factors. However, none of the included ATC patients had a medical history of cardiovascular diseases. Moreover, in our study, age did not correlate significantly with plasma PTX3 concentrations. Other limitations include the small sample size, particularly for ATC given its rarity, as well as the heterogeneity of the cohort and the resulting inability to perform comprehensive analyses across all groups, histologic subtypes, or AJCC stages. The absence of a healthy control cohort further restricts the interpretation of circulating PTX3 levels.

## 4. Materials and Methods

### 4.1. Study Design and Population

In this prospective observational cohort study, we included two sets of consecutive patients: a group of patients with various histologic subtypes of TC (papillary, follicular, oncocytic, PDTC, and ATC), and a control group consisting of patients with benign thyroid pathology (chronic autoimmune thyroiditis and multinodular goiter).

The research protocol was approved by the Ethics Committee of The National Institute of Endocrinology “C. I. Parhon” (No. 08/20.04.2022) and Radboud University Medical Center, Nijmegen, the Netherlands (2022-16025 & 2017-3628). All patients provided informed consent for participation.

The TC group included patients who were either newly diagnosed with TC or who were under regular follow-up for recurrent disease with locoregional or distant metastases in two tertiary centers—National Institute of Endocrinology “C. I. Parhon”, Bucharest, Romania, and Radboud UMC, Nijmegen, the Netherlands. Blood samples were collected prospectively between 2018 and early 2023 before surgical resection in newly diagnosed TC patients or during a follow-up visit in patients with structurally recurrent TC.

The patients in the control group were selected at the National Institute of Endocrinology C. I. Parhon, Bucharest, between November 2022 and June 2023, based on their medical records, euthyroid status, thyroid gland in situ, benign pathology on cytology, no suspicious ultrasound characteristics ultrasound, and assigned to the control group after total thyroidectomy, matched by age and sex with the TC patients.

Patients with Graves’ disease, chronic systemic inflammatory diseases, other active neoplasms, active known infections, major cardiovascular disease (e.g., history of myocardial infarction, stroke, or peripheral artery disease), medication interfering with the immune system, systemic anti-cancer treatment, and surgery <3 months before blood withdrawal, liver or renal failure, self-reported alcohol consumption of >21 units per week or pregnancy were excluded.

Patients’ demographics, type and extent of surgical resection, pathology reports, and disease staging were collected from electronic medical records and primary data collection.

### 4.2. Definitions, Outcome, and Measurements

In patients with lymphocytic thyroiditis, the diagnosis was documented by high anti-TPO serum concentrations or histologic confirmation after surgery.

All patients with TC had active structural disease at the time of blood collection. The extent of TC was classified according to the American Joint Committee on Cancer staging system for differentiated and poorly differentiated thyroid cancer, 8th edition/TNM Classification System [26]. Radioiodine resistance (RAIR) was defined according to previously proposed criteria [27].

### 4.3. PTX3 Concentration in Circulation and Tissue Expression

Plasma PTX3 concentrations were measured using enzyme-linked immunosorbent assay (ELISA) in blood samples collected in EDTA vacutainers from fasting patients in the morning. Samples were centrifuged at 3000× *g* for 15 min within one hour of collection and stored at −80 °C until analysis. PTX3 expression in tissue was assessed by immunohistochemistry (IHC) on paraffin-embedded samples. Single and double staining were performed to evaluate PTX3 expression and its potential co-localization with CD68-positive macrophages (Supplementary Table S1).

PTX3 immunoreactivity was evaluated on 3,3′-diaminobenzidine (DAB) stained sections, with brown cytoplasmic and extracellular staining considered specific. Staining intensity in tumor areas was scored semi-quantitatively as negative, positive, or strongly positive, based on the overall visual impression of signal intensity. CD68 staining was interpreted similarly using its chromogenic substrate (Supplementary File S1). All slides were assessed by the same experienced endocrine pathologist to ensure consistency.

Given the retrospective design of the study and the rarity of ATC and PDTC, the number of available tissue samples for these subtypes was limited. The analysis of PTX3 and CD68 staining in ATC and PDTC was therefore descriptive, as the small sample size precluded meaningful statistical comparisons.

### 4.4. Statistical Analysis

In the main analysis, plasma PTX3 concentrations were compared between patients with benign thyroid disease and TC, and between subgroups of TC patients based on clinicopathological factors. Normality was assessed using the Kolmogorov–Smirnov test. Parametric (*t*-test, ANOVA) and non-parametric tests (Mann–Whitney, Kruskal–Wallis) were used as appropriate. Correlations between PTX3 concentrations and demographic variables were evaluated using Pearson’s or Spearman’s correlation tests. A *p*-value < 0.05 was considered statistically significant. Statistical analysis was conducted using GraphPad Prism version 8.0.2 (Supplementary File S1).

## 5. Conclusions

In conclusion, this study provides novel insights into the expression and potential role of PTX3 in ATC. While overall no significant difference in plasma PTX3 levels was observed between patients with TC and benign thyroid disease, increased PTX3 expression was found in patients with ATC, suggesting its potential association with the aggressive clinical phenotype of this cancer subtype. The immunohistochemistry experiments were inconclusive regarding the origin of the PTX3 secretion. Despite the limitations of this study, the findings suggest that PTX3 may be a potential contributor to the inflammatory microenvironment in ATC. Future research should focus on validating these findings in larger cohorts and utilizing advanced technologies to further explore the mechanistic pathways of PTX3 in the immunologic landscape of aggressive thyroid cancer.

## Figures and Tables

**Table 1:** Clinical and pathological characteristics of the patients with thyroid cancer and the corresponding Pentraxin 3 (PTX3) concentration.

		No	PTX3 (pg/mL) *	*p*-Value
Age at diagnosis (years)				*p* = 0.301
<55		25	86.3 ± 23.7	
≥55		30	78.6 ± 20.1	
Plasma collected before surgery (DTC)		37	73.7 (48.2, 127.2)	*p* = 0.548
Plasma collected after primary surgery—recurrent active disease (DTC)		14	80.4 (44.7, 261.7)	
DTC (all)		42	76.6 (69.3, 103.2)	*p* = 0.392 **
	Classic PTC	36	75.5 (69.98, 94.6)	
	Tall cell PTC	6	73.3 (54.1, 108.2)	
	FTC	3	72.6 (65.8, 89.84)	
	OTC	4	71.2 (64.5, 83.5)	
PDTC		2	155.5 (49.3, 261.7)	
ATC		4	269.4 (224.2, 321.4)	
Multifocality ^a^		24	80.5 (65.4, 93.8)	*p* = 0.577
No multifocality ^a^		13	84.5 (84.5, 20.6)	
RAIR ^b^		11	84.6 (74.9, 115.1)	*p* = 0.739
Non-RAIR ^b^		40	74.9 (69.9, 94.6)	
Distant metastases		11	84.6 (59.7, 115.1)	*p* = 0.645
Without distant metastases		32	73.1 (66.8, 100.1)	
AJCC stage	Stage I	22	81.5 ± 21.95	*p* = 0.040
	Stage II	16	76.74 ± 22.4	
	Stage III	7	121 ± 62.88	
	Stage IV	10	165.4 ± 103.1	

PTC: papillary thyroid cancer, FTC: follicular thyroid cancer, OTC: oncocytic thyroid cancer, DTC: Differentiated thyroid cancer, PDTC: poorly differentiated thyroid cancer, ATC: anaplastic thyroid cancer, RAIR: radioiodine resistance. ^a^ Included DTC only. ^b^ Included DTC, PDTC, ATC patients; staging was performed for all patients according to AJCC 8th ed. * Note: Values are expressed as mean ± standard deviation (SD) or median (interquartile range, IQR). ** Compared classic PTC vs. aggressive variants of PTC.

## Data Availability

The original contributions presented in this study are included in the article/Supplementary Material. Further inquiries can be directed to the corresponding author(s).

## Supplementary Materials

The following supporting information can be downloaded at: https://www.mdpi.com/article/10.3390/ijms262311335/s1.

## Abbreviations

The following abbreviations are used in this manuscript:

AJCC	American Joint Committee on Cancer
ATC	Anaplastic Thyroid Carcinoma
BMI	Body Mass Index
CD68	Cluster of Differentiation 68 (Macrophage Marker)
DAMPs	Damage-Associated Molecular Patterns
DTC	Differentiated Thyroid Cancer
ELISA	Enzyme-Linked Immunosorbent Assay
IL-1β	Interleukin-1 Beta
IL-6	Interleukin-6
IHC	Immunohistochemistry
PDTC	Poorly Differentiated Thyroid Carcinoma
PRMs	Pattern Recognition Molecules
PTC	Papillary Thyroid Cancer
PTX3	Pentraxin 3
RAIR	Radioiodine Resistance
TC	Thyroid Cancer
TAM	Tumor-Associated Macrophage
TLR	Toll-Like Receptor
TNF	Tumor Necrosis Factor
TSG-14	Tumor Necrosis Factor-Stimulated Gene 14
