# Supplementary material for: High Local and Systemic Expression of Pentraxin-3 in Anaplastic Thyroid Cancer
Source: Int J Mol Sci. 2025 Nov 24;26(23):11335. doi: 10.3390/ijms262311335 (PMC12692025; doi:10.3390/ijms262311335)
Supplement: Supplementary file 1 [file ijms-26-11335-s001.zip › ijms-3985047-supplementary.pdf]

Supplementary Table S1: Standard immunostaining protocol for paraffin-section soft tissues: PTX3 and CD68 double staining

Supplementary Table S2: PTX3 and CD68 expression in TC tissues (summary of IHC findings).

Supplementary File S1. Methodology: PTX3 plasma measurement, immunohistochemical assessment, and statistical analysis

Supplementary Table S1. Standard immunostaining protocol for paraffin-section soft tissues: PTX3 and CD68 double staining

Standard immunostaining protocol for paraffin-section soft tissues
PTX3 and CD68 double staining

Materials and protocol

- Xylol and ethanol: 5 min each step; refresh the 1st xylol before start and switch this one with the 2nd Xylol; the 2nd Xylol must be placed 1st; refresh also 100% ethanol
- Wash buffer PBS-Tw 0.05%:
  200 ml PBS 10x + 1,8L H_2O from tap with green/white handle + 1ml Tween-20
- Antigen retrieval: Tris-EDTA pH9 (Tris 1,21g + EDTA 0,37g + H2O 1L (pH=9))
- 3% H_2O_2 solution: 180 ml PBS or Methanol + 20 ml H_2O_2 30%: make it just before you need it! (H_2O_2 30% Merck 1.07210.0250)
- Antibody buffer: 1% BSA in PBS (Bovine Albumin Sigma **A-9647**; make this in a glass bottle and do not shake the solution)
- Antibody solutions:
  - Gt a Rb-BIOT Vector PK-6101 1:400 in 1% BSA/PBS
  - ABC-HRP Vector PK-6101 1:200 in PBS
    Make this solution at least 30 min before use and mix it well.
  - BrightVision-poly-AP Immunologic DPVO55AP undiluted
- DAB solution: 1 ml DAB ([10mg/ml] (3,3'-diaminobenzidine Sigma D-5637) + 9 ml DAB-buffer (Tris-HCl 50mM pH7,6) + 10 µl H_2O_2. Keep the DAB solution in the dark and add the H_2O_2 just before use. Develop for 10 minutes.
- BrightVision-poly-AP kit: SK-5400 BCIP/NBT blue:
  5 ml 0.1M Tris-HCL ph9.5 + 2 drops solution 1 + 2 drops solution 2 + 2 drops solution 3

Method:

1. Paraffin embedded thyroid tissue was cut at 4 µm and mounted on Superfrost™ Plus Microscope Slides.
2. Sections were dewaxed in: xylol – xylol – 100% - 96% - 70% ethanol - 5 min at rT each step
3. Rinse in water - 1x
4. Antigen-retrieval in Tris-EDTA (200 ml in green box with gray rack)
   - 2 min in microwave full power (700w), 10 min in rice cooker, 30 min to cool down at rT

5. Rinse in PBS-Tween 0.05% 1x
6. **Block endogenous peroxidase** with 3% H2O2 in PBS for 15 min at rT
7. Rinse in PBS-Tween 0.05% 1x
8. Circle with DAKO-pen around each section, do not let the tissue dry!
9. **Block the unspecific sites** with 20% normal goat serum (NGS)+**PBS** for 30 min rT in humid chamber.
10. Drain the slides and incubate 1 hr with **Rabbit polyclonal antibody anti Human PTX3** diluted 1:250 in 1% BSA in PBS in humid chamber
11. Rinse in PBS-Tween 0.05% 3x
12. 2nd antibody Goat anti Rabbit-BIOT 1:400 in 1% BSA in PBS – 30 min at rT
13. Rinse in PBS-Tween 0.05% 3x
14. 3rd antibody ABC-HRP 1:200 in PBS – 30 min at rT
15. Rinse in PBS-Tween 0.05% 3x
16. Add 250 µl of DAB solution on each section - 10 min at rT
17. Rinse in PBS-Tween 0.05% 3x

Continue with the double staining:

18. Incubate 1 hr at rT with **Mouse monoclonal antibody anti Human CD68** diluted 1:50 in 1% BSA in PBS in humid chamber
19. Rinse in PBS-Tween 0.05% 3x
20. 2nd antibody BrightVision-poly-AP solution - 30 min at rT
21. Rinse in PBS-Tween 0.05% 3x
22. Add BCIP/NBT blue solution on each section - 30min at 37°C
23. Rinse in PBS-Tween 0.05% 1x
24. Rinse in tap water 2x
25. Dip in Hematoxylin – 1 sec
26. Rinse in running tap water – 10 min at rT
27. PTX3 single: Dehydrate: 70% - 96% - 100% - xylol – xylol - 5 min at rT each step; Enclose in Permount
28. CD68 and PTX3+CD68: Enclose in Gelatin/Glycerin! Do not dehydrate these slides

rT: room temperature

Supplemental Table S2. PTX3 and CD68 expression in TC tissues (summary of immunohistochemistry findings).

Histology		*Age, sex*	*PTX3 staining*	*CD68 staining*
Goiter				
	G1	73, F	Negative	Negative
	G2	62, F	Negative	Negative
PTC		54, M	Negative	Negative
ATC				

ATC1	47, F	Strongly positive	Strongly positive
ATC2	81, F	Strongly positive	Positive
ATC3	70, M	Strongly positive	Positive
ATC4	75, M	Negative	Positive

PTC: papillary thyroid cancer, ATC: anaplastic thyroid cancer, F: female, M: male

Somatic mutations assessed: *BRAF V600E* in ATC1; *TP53,* and *NRAS* in ATC2; *PDGFRB*, *PTEN*, and *CDKN2A* in ATC3; and *CDKN2A* in ATC4.

Supplementary File S1. Methodology: PTX3 plasma measurement, immunohistochemical assessment, and statistical analysis

Plasma PTX3 Measurement

Blood samples were collected in EDTA vacutainers from fasting patients in the morning. Samples were centrifuged at 3000g for 15 minutes within one hour of collection, and plasma was stored at -80°C until analysis. Plasma PTX3 concentrations were determined using enzyme-linked immunosorbent assay (ELISA) kits (Quantikine™ ELISA Human Pentraxin 3/TSG-14 Immunoassay, R&D Systems, Catalog Number DPTX30B), following the manufacturer's instructions.

Immunohistochemical Analysis of PTX3 and CD68 Expression

PTX3 expression in tissue was evaluated in paraffin-embedded samples from patients with papillary thyroid carcinoma (PTC), anaplastic thyroid carcinoma (ATC), and benign goiter. Immunohistochemistry (IHC) was performed using polyclonal rabbit anti-human PTX3 antibodies (Sigma-Aldrich, HPA069320) at a dilution of 1:250. To assess potential co-localization of PTX3 in tumor-infiltrating macrophages, CD68 staining was performed using monoclonal mouse anti-human CD68 antibodies (Biorad, MCA1815) at a dilution of 1:50.

Both single and double staining techniques were applied. PTX3 visualization was achieved using 3,3'-diaminobenzidine (DAB) chromogen (brown staining), while CD68 positivity was detected using Fast Red Chromogen (fuchsia staining) and Alkaline Phosphatase (violet staining). Sections were counterstained with hematoxylin. Normal thyroid tissue was used as a control for baseline expression, and a buffer solution served as a negative control. The analysis focused on both extracellular stromal and intracellular PTX3 expression in tumor tissues.

Statistical Analysis

Data were presented as numbers and percentages, means and standard deviations (SD), or medians and interquartile ranges, as appropriate. Comparisons of mean plasma PTX3 levels

were performed between benign thyroid disease and thyroid carcinoma (TC) patients, including presence vs absence of lymphocytic thyroiditis in both groups. Within the TC cohort, subgroup analyses included age (<55 vs ≥55 years), multifocality, recurrent vs treatment-naive disease, aggressive vs non-aggressive PTC subtypes, PDTC and ATC vs differentiated TC, RAIR vs non-RAIR, AJCC staging, and distant metastases.

Normality testing was conducted using the Kolmogorov-Smirnov test. If data were normally distributed, including after logarithmic transformation, a t-test was used for two-group comparisons; otherwise, a Mann-Whitney test was applied. For comparisons involving more than two groups, ANOVA or the Kruskal-Wallis test was used based on data distribution. Correlations between PTX3 levels and demographic characteristics (age, BMI, sex) were assessed using Pearson's correlation for normally distributed data and Spearman's correlation for non-normally distributed data. A p-value < 0.05 was considered statistically significant. Statistical analysis was conducted using GraphPad Prism version 8.0.2.
